# Development and validation of an index system for family caregivers’ capacity in home-based disabled elderly care

**DOI:** 10.1093/geroni/igaf122.2421

**Published:** 2025-12-31

**Authors:** Shujing Suo, Yun Li

**Affiliations:** Zhengzhou University, Zhengzhou, Henan, China (People’s Republic); Zhengzhou University, Zhengzhou, Henan, China (People’s Republic)

## Abstract

**Background:**

Family caregivers’ capacity affects both disabled elders’ health and their own experience. However, there is currently a lack of tools to assess the capacity of family caregivers of older adults with disability at home. This study aimed to establish and validate an index system of the caregiving capacity of family caregivers of the elderly with disabilities at home.

**Methods:**

A Literature review, cross-sectional survey, and qualitative interviews were used to construct an index pool. The Delphi method was used to establish the index system, and the Analytic Hierarchy Process was used to determine the weight of the indicators. Finally, the index system was transformed into a questionnaire, and a survey was conducted to validate the reliability and validity of the indicator system.

**Results:**

The effective response rate for the two rounds of expert consultation was 100%. After two rounds, 3 first-level indicators, 6 second-level indicators, and 22 third-level indicators were determined. The coefficient of expert authority for both rounds is 0.842. The first, second, and third-level indicators of Kendall’s W consistency coefficient were 0.650, 0.579, and 0.257 P<0.001. The overall Cronbach’s α coefficient of the index system was 0.941, and the content validity index of the index item level ranged from 0.833 to 1.000.

**Conclusions:**

The index system of family caregivers of the elderly with disabilities at home is comprehensive, scientific, and reasonable, which can provide a reference for the development of education or training of family caregivers in the future and the evaluation of its effect.

